# Long-term Blood Pressure Variability and Cerebrovascular Changes on CT in a Community-based Elderly Population

**DOI:** 10.2188/jea.11.190

**Published:** 2007-11-30

**Authors:** Eri Tsukishima, Hiroya Saito, Koichi Shido, Gen Kobashi, Gong Ying-Yan, Reiko Kishi, Minehisa Niino, Kiyotaro Kondo, Iwao Sugimura

**Affiliations:** 1 Department of Public Health, Hokkaido University Graduate School of Medicine.; 2 Department of Radiology, Asahikawa Kosei General Hospital.; 3 Department of Social Services, Graduate School of Health Sciences, University of Hokkaido.; 4 Department of Health for Senior Citizens, Hokkaido University Graduate School of Medicine.; 5 Department of Health Examination, Asahikawa Kosei General Hospital.; 6 University of the Air.; 7 The Director of Asahikawa Kosei General Hospital.; 8 Sapporo City Kiyota Health Center.

**Keywords:** silent infarcts, leukoaraiosis, computed tomography, blood pressure, elderly, longitudinal study

## Abstract

The effects of long-term blood pressure (BP) levels on cerebrovascular changes were analyzed in a community-based healthy elderly population. Cranial computed tomography (CT) was performed for 300 residents aged 69 years and older. Long-term BP during the ten years prior to CT was assessed, and the cerebrovascular changes were compared among different patterns of long-term blood pressure variability. White matter lesions (WML) and/or silent infarctions (SI) were found in 73 subjects (23.6%). Multiple logistic regression analysis showed that subjects with long-term diastolic hypertension (DHT) had the highest risk of cerebrovascular changes (adjusted odds ratio (OR), 95% confidence interval (CI); 7.1, 2.4-21.6, for WML; 7.2, 2.7-19.4, for SI), and that long-term isolated systolic hypertension (ISHT) was significantly associated with SI (adjusted OR, 95%CI, 2.3, 1.1-4.9), but not with WML (adjusted OR, 95%CI, 1.3, 0.5-3.3). Efforts to prevent both DHT and ISHT would be beneficial, though different underlying mechanisms for WML and SI were suggested.

## INTRODUCTION

Hypertension is one of the most important risk factors associated with cerebrovascular changes such as periventricular white matter lesions (WML) and silent cerebral infarctions (SI)^1^^, ^^2^^)^. These changes are frequently detected in the elderly persons using computed tomography (CT) or magnetic resonance imaging (MRI), and appear to be associated with cognitive decline^3^^, ^^4^^, ^^5^^)^, neurological damage^6^^, ^^5^^, ^^7^^)^, and a high risk for clinical stroke^8^^)^.

The relation between hypertension and cerebrovascular changes has been well demonstrated. History of hypertension was significantly associated with WML in community-based elderly populations^1^^, ^^2^^)^, and with SI among stroke patients^9^^)^. WML was also associated with higher blood pressure that measured cross-sectionally^1^^)^. Additionally, subjects with lacunar infarcts showed higher ambulatory blood pressure than those with fewer or no lacunae^10^^)^.

As for short-term blood pressure variation, several studies using a non-invasive 24-hour blood pressure recorder suggested that absence of nocturnal fall or extreme variation in ambulatory blood pressure were associated with cerebrovascular changes^11^^, ^^12^^, ^^13^^)^. However, long-term blood pressure variability in hypertensive subjects has not been clearly examined in relationship to cerebrovascular changes. A 3-year longitudinal study on a community-based population showed that “prevalent hypertension subjects” that were previously identified as hypertensive had a higher risk of WML on MRI^14^^)^.

Since 1975, a health check-up screening program has been performed for adult residents every year in a rural town of Takasu in Hokkaido, Japan. The present study examined variability of blood pressure over ten years from the longitudinal data bank of the program in relation to subclinical changes that appeared on CT in the aging brain. We also investigated whether the occurrence of these changes differed among subtypes of long-term hypertension subjects.

## SUBJECTS AND METHODS

### Study Population

The target population for this study was all healthy elderly residents in the agricultural town Takasu, Japan. Cranial CT scanning was planned for the healthy residents among the entire population aged 69 years and older, and performed between December 1991 and January 1998 for 388 elderly individuals. The response rate was estimated at nearly 44%, which was a rate of participants among residents aged 69 years and older in 1991 and those became 69 years between 1992 and 1998. Since the objective of our study was to investigate the subclinical changes, the following residents were excluded: 43 persons who were functionally disabled or had a neurological disorder, nine who had a history of stroke, and nine who had a definite abnormality on cranial CT. The abnormal findings detected on CT included an intracranial tumor, an extradural hydroma, and a large infarction (> 30mm). To obtain adequate data on long-term blood pressure variability, we included only residents who had had health check-ups for more than five years before CT scanning. Thus the final sample included 300 residents (145 men and 155 women, mean age 72.9 years, standard deviation (SD) 3.9 years). Characteristics of all subjects are shown in Table 1, 2.

**Table 1.** Characteristics of subjects enrolled in the study.

Characteristics	Mean (SD) or number (%)	
Mean age (years)	72.9	SD 3.9
Male subjects	145	48.3%
Lower education (< 6 years)	118	40.0%
Baseline lifestyles		
Cigarette smoking	59	20.1%
Alcohol drinking (> 3 times a week)	112	38.2%
Self-reported medical history		
Heart disease	62	21.0%
Renal disease	22	7.4%
Diabetes	33	11.0%
Respiratory disease	30	10.0%
Hypertension	114	38.3%

**Table 2.** Measures of long-term blood pressure during the ten-year period of subjects enrolled in the study.

Measures of long-term blood pressure	Mean	SD
Mean SBP (mmHg)	135.9	15.2
Within-subject CV in SBP	9.9	3.1
Gain in SBP per year (mmHg)	1.1	2.7
Mean DBP (mmHg)	78.4	8.3
Within-subject CV in DBP	10.4	3.9
Gain in DBP per year (mmHg)	0.12	1.7
Mean PP (mmHg)	57.4	10.9

Values were calculated from longitudinal data bank over ten years. BP systolic blood pressure, CV coefficient of variation, DBP diastolic blood pressure, PP pulse pressure.

### Health examination

Records of longitudinal data were obtained from the data bank of the health check-up screening program. This program had been performed for all residents who underwent CT scanning, and included physical measurements, interviews concerning smoking and drinking status, routine blood tests, electrocardiograms, and medical and neurological examinations.

Blood pressure, and smoking and drinking habits were analyzed for ten years prior to CT scanning. For residents who had participated in health check-up examinations two or more times, the earliest examination in the ten-year term was analyzed as the baseline examination. The mean frequency of participation during the ten-year period was 7.8 times (SD 2.4), and the average interval between the baseline examination and CT scanning was 9.0 years (SD 1.1).

### Long-term blood pressure variability

Blood pressure was measured annually at the check-up examinations. It was measured in the sitting position early in the morning after 5 minutes rest, using a regular sphygmomanometer. Blood pressure was also measured in the same way in the morning on the day of CT scanning.

As measures of the long-term blood pressure variability over ten years, the following values were analyzed in each subject for both systolic blood pressure (SBP) and diastolic blood pressure (DBP); value at baseline, value at the day of CT, mean value during the ten-year term, coefficient of variation (CV) in blood pressure during the term, and gain in blood pressure per year. Gain in SBP per year was defined as; gain in SBP/y = ((SBP at CT) - (SBP at baseline)) / (interval years). As measures of the pulse pressure (PP) variability, value at baseline, value at the day of CT, and mean value during the term were analyzed.

**Figure 1.**
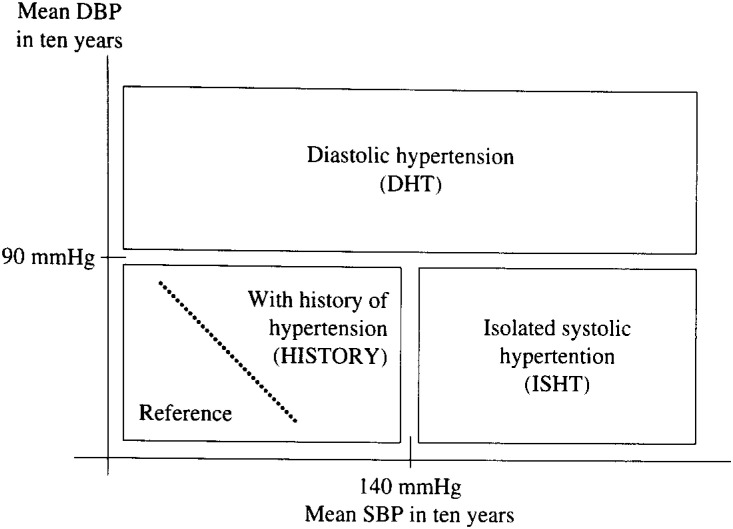


To assess the effect of long-term blood pressure levels, subjects were sorted by average level of blood pressure over ten years, using the values in the criteria for hypertension of the Joint National Committee^15^^)^ as the division values of long-term blood pressure (Fig. 1). The subtype of diastolic hypertension (DHT) included subjects with a mean DBP values greater than or equal to 90 mmHg, the subtype of isolated systolic hypertension (ISHT) included those with a mean SBP greater than or equal to 140 mmHg and a mean DBP of less than 90 mmHg. In the subjects with a mean SBP of less than 140 mmHg and a mean DBP of less than 90 mmHg, subjects with a history of hypertension (HISTORY) were divided from those without a history, because their blood pressure had stayed in the appropriate levels over ten years because of pharmacological or non-pharmacological treatment. A hypertension group (All HT) was defined as a group of subjects with a mean SBP of greater than or equal to 140 mmHg or a mean DBP of greater than or equal to 90 mmHg or a history of hypertension. Thus, All HT consisted of three subtypes; DHT, ISHT, and HISTORY. Prevalence of cerebrovascular changes in each subtype of hypertension was compared with a reference group of subjects with a mean SBP of less than 140 mmHg and a mean DBP of less than 90 mmHg and without a history of hypertension.

### Cranial CT

Axial CT scans were obtained with 10-mm-thick sections. The same experienced radiologist (H. S.), who was blinded to the clinical information, examined all the CT scans. In each subject, white matter lesions (WML) and silent infarct (SI) were graded into four categories. WML were assessed by grading the extent of low-density area in the periventricular white matter, 1, no detectable change; 2, thin lining low-density area; 3, irregular low-density area within an inner half area of the white matter; 4, low-density area extending into the outer half area of the white matter around the lateral ventricle. SI were rated as follows; 1, no detectable low-density foci; 2, single, small foci; 3, several infarcts; 4 multiple infarcts. Each change was dichotomized to avoid cells with small numbers. WML were defined as absent if the grading was 1, and as present if the grading was 2 to 4. The definition of the classification of SI was the same. In order to assess the reliability of the ratings, two experienced radiologists independently graded 160 CT scans out of the 300 scans, and the interrater reliabilities of the classifications were high enough (Kappa = 0.92 and 0.88, for WML and SI, respectively).

On the same day of CT scanning, trained public health nurses obtained demographic characteristics, lifestyles, and self-reported daily function via interview. Medical histories were also interviewed about heart diseases, renal diseases, diabetes, respiratory diseases and hypertension. Physical and neurological examinations including blood-pressure measurement were performed on the same day.

### Statistical Analysis

The presence of cerebrovascular changes on CT was analyzed and assigned to three groups; WML without SI (WML(+)SI(-)), SI without WML (WML(-)SI(+)), and both WML and SI (WML(+)SI(+)), compared with a seemingly normal group (WML(-)SI(-)). Frequencies of cerebrovascular findings were compared between subjects with and without potential risk factors. To estimate an effect of each factor on cerebrovascular changes, age-adjusted odds ratios (OR) with their 95% confidence intervals (CI) were calculated by logistic regression estimation.

Continuous measures of long-term blood pressure variability were compared among four groups of subjects with and without cerebrovascular changes, after adjusting for age by analysis of variance.

Differences in prevalence of WML(+)SI(-), WML(-)SI(+), and WML(+)SI(+), were compared among sorted subtypes of long-term blood pressure. Compared with reference group, age-adjusted OR of DHT, ISHT, and HISTORY were calculated by logistic regression analysis.

Multiple logistic regression was used to analyze effects of patterns of long-term blood pressure on WML and on SI separately. Subtypes of long-term blood pressure were compulsorily entered into the model, and age, the most important confounding factor, was also entered. Other potential confounding factors, such as sex, education, lifestyles, and medical histories were considered and selected into the model by stepwise analysis. We used the computer software package Statistical Analysis System (SAS) ver. 6.

## RESULTS

### CT findings and baseline examination

Among 300 elderly subjects, WML were detected in 32 subjects, and SI in 52 subjects. Neither WML nor SI was found in 233 subjects (78.2%), while fifteen subjects (5.1%) had WML without SI (WML(+)SI(-)), 35 subjects (11.9%) had SI without WML (WML(-)SI(+)), and seventeen (4.8%) had both WML and SI (WML(+)SI(+)).

Frequencies of CT findings and age-adjusted odds ratios (OR) are shown in Table 3. Subjects with cerebrovascular changes were likely to be older (mean age = 74.6, 73.4, and 74.1 years in WML(+)SI(-), WML(-)SI(+), and WML(+)SI(+); compared with 72.6 in WML(-)SI(-)). Lower education was significantly associated with SI (adjusted OR = 2.5, 95% confidence interval (CI) 1.2-5.2). Subjects who had a smoking habit at the baseline examination tended to have more cerebrovascular changes. The frequency of WML(+)SI(+) among subjects with smoking habit was significantly higher than without (adjusted OR = 3.5, 95%CI 1.1-10.7).

**Table 3.** Frequencies and age-adjusted odds ratios for each CT finding among subjects with potential risk factors.

		WML(-) SI(-)	WML(+) SI(-)		WML(-) SI(+)		WML(+) SI(+)	
			
		No %	No %	Adjusted OR (95%CI)	No %	Adjusted OR (95%CI)	No %	Adjusted OR (95%CI)
All subjects	n=300	233	15		35		17	
		78.2	5.1		11.9		4.8	
Socio-demographical factors								
Mean age (mean±standard deviation)		72.6±3.3	74.6±3.3		73.4±3.4		74.1±5.6^a)^	
Male sex	n=145	112	7	0.8	15	0.7	11	1.7
		77.2	4.8	(0.2-2.0)	10.3	(0.3-1.4)	7.6	(0.6-5.0)
Lower education	n=118	85	5	0.9	21	2.5	7	1.1
		72.0	4.2	(0.3-2.8)	17.8	(1.2-5.2)	5.9	(0.4-0.3)
Baseline lifestyles								
Smoking	n=59	40	5	2.1	8	1.4	6	3.5
		67.8	8.5	(0.6-6.4)	13.6	(0.5-3.1)	10.2	(1.1-10.7)
Drinking (>3 times a week)	n=112	84	6	1.2	16	1.5	6	1.3
		75.0	5.4	(0.4-3.5)	14.3	(0.7-0.3)	5.4	(0.4-3.9)
Subjective medical history								
Heart diseases	n=62	44	2	0.5	10	1.6	6	2.2
		71.0	3.2	(0.1-2.1)	16.1	(0.7-3.5)	9.7	(0.7-0.6)
Renal diseases	n=22	15	1	0.9	4	1.8	2	1.8
		68.2	4.6	(0.05-5.2)	18.1	(0.5-5.4)	9.1	(0.3-7.4)
Diabetes	n=33	24	2	1.2	4	1.1	3	1.5
		72.7	6.1	(0.2-4.7)	12.1	(0.3-3.0)	9.1	(0.3-5.3)
Respiratory diseases	n=30	26	0	-	4	1.0	0	-
		86.7	0	-	13.3	(0.3-2.8)	0	-
Hypertension	n=114	76	4	0.7	21	3.0	13	6.4
		66.7	3.5	(0.2-2.2)	18.4	(1.5-6.4)	11.4	(2.2-23.3)

WML, white matter lesion; SI, silent infarction; Adjusted OR, Odds ratio of each factor compared with reference category of WML(-)SI(-) after adjusting for age; 95%CI, 95% confidence interval; a) p<0.05 compared with WML(-) SI(-), using Tukey’s multiple comparison.

With regard to self-reported medical histories, the frequencies of cerebrovascular changes among subjects with a history of hypertension were significantly higher, and the age-adjusted odds ratio for WML(+)SI(+) was 6.4 (95%CI 2.2-23.3).

### Long-term blood pressure variability and cerebrovascular changes detected on CT

Several measures for long-term blood pressure variability were assessed to determine if they had any associations with cerebrovascular changes on CT (Table 4). All measures for levels of long-term SBP, such as SBP at CT, SBP at baseline, and mean SBP over ten years, were higher in subjects with cerebrovascular changes than in those without. After adjusting for age, the highest value of mean SBP was found in subjects with both WML and SI (age-adjusted mean = 147.8 mmHg), which was significantly different from the reference group (134.6 in WML(-)SI(-)). Otherwise, measures for variation in long-term SBP such as within-subject CV and gain in SBP per year were not significantly different among CT finding groups.

**Table 4.** Differences in age-adjusted means of long-term BP variability between subjects with each CT findings.

	WML(-) SI(-)	WML(+) SI(-)	WML(-) SI(+)	WML(+) SI(+)
Number of subjects	233	15	35	17
Measures of long-term SBP during the ten-year period				
Mean SBP	134.6	136.1	138.2	147.8 ^a)^
Within-subject CV in SBP	9.9	10.6	9.9	9.3
Gain in SBP per year	1.2	1.7	0.6	0.7
Measures of long-term DBP during the ten-year period				
Mean DBP	77.5	80.4	79.0	88.3 ^a) b) c)^
Within-subject CV in DBP	10.5	9.7	10.2	10.1
Gain in DBP per year	0.1	0.7	0.4	-0.4
Measures of long-term PP during the ten-year period				
Mean PP	57.1	55.6	59.3	59.6

Values are age-adjusted mean. WML, white matter lesion; SI, silent infarction; SBP, systolic blood pressure; CV, coefficient of variation; DBP, diastolic blood pressure; PP, pulse pressure.

^a)^, ^b)^, ^c)^, p<0.05 compared with WML(-)SI(-), WML(+)SI(-), and WML(-)SI(+), respectively, using Tukey’s multiple comparison.

As for long-term DBP, similar associations were shown. All measures for DBP levels were higher in subjects with cerebrovascular changes. The age-adjusted mean DBP was the highest in subjects with both WML and SI (88.3 mmHg), and significantly higher than each of the other three groups (77.5, 80.4, and 79.0 in WML(-)SI(-), WML(+)SI(-) and WML(-)SI(+), respectively). Measures for long-term variation in DBP were not significantly different among the four groups.

Mean PP during ten years tended to be higher in subjects with cerebrovascular changes, and the highest in subjects with both WML and SI. However, the difference was not statistically significant.

### Cerebrovascular changes in subtypes of long-term blood pressure pattern

Among subtypes of long-term hypertension, there were some differences in frequencies of cerebrovascular changes (Table 5). Subjects with high long-term DBP (DHT; long-term DBP > 90 mmHg) had the highest risk of cerebrovascular changes. The risk of both WML and SI was the highest among subtypes of long-term blood pressure, and the age-adjusted odds ratio was 15.2 (95%CI 4.9-49.2). Subjects in the DHT group also had the highest risk of WML without SI, and SI without WML, though they were not significantly different from the other subtypes. The age-adjusted OR were 2.4 for WML(+)SI(-) and 2.6 for WML(-)SI(+). In the ISHT subtype, in which subjects had high long-term SBP (mean SBP > 140 mmHg) and low DBP (mean DBP < 90 mmHg), more SI were found compared with the reference group (age-adjusted OR = 1.8). Six persons had WML without SI (age-adjusted OR = 1.4), and four had both WML and SI (age-adjusted OR = 0.6). In the HISTORY subtype, in which subjects had a history of hypertension and appropriate level of long-term BP, no persons had WML without SI, four had SI without WML, and only one person who had both WML and SI. Subjects in the HISTORY were likely to have more SI, but the differences were not significant. Subjects in the all HT group, who had a history of hypertension and/or high blood pressure had significantly more cerebrovascular changes. The age-adjusted odds ratios were 1.4 (95%CI 0.5-4.1), 3.1 (95%CI 1.5-7.0) and 3.9 (95%CI 1.3-14.1) for WML(+)SI(-), WML(-)SI(+) and WML(+)SI(+), respectively (Table 5).

**Table 5.** Frequencies and age-adjusted odds ratios for each CT finding among subjects with hypertension.

		WML(-) SI(-)	WML(+) SI(-)		WML(-) SI(+)		WML(+) SI(+)	
			
		No %	No %	Adjusted OR (95%CI)	No %	Adjusted OR (95%CI)	No %	Adjusted OR (95%CI)
DHT	n=30	15	2	2.4	5	2.6	8	15.2
		50.0	6.7	(0.5-4.1)	16.7	(0.8-7.4)	26.7	(4.9-49.2)
ISHT	n=100	74	6	1.4	16	1.8	4	0.6
		74.0	6.0	(0.5-4.1)	16.0	(0.9-3.7)	4.0	(0.2-1.8)
HISTORY	n=20	15	0	–	4	1.8	1	0.9
		75.0	0	–	20.0	(0.5-5.3)	5.0	(0.05-4.9)

All HT	n=150	104	8	1.4	25	3.1	13	3.9
		69.3	5.3	(0.5-4.1)	16.7	(1.5-7.0)	8.7	(1.3-14.2)

Reference	n=150	129	7	1.0	10	1.0	4	1.0
		86.0	4.7		6.7		2.7	

WML, white matter lesion; SI, silent infarction; Adjusted OR, Odds ratio of each factor compared with reference category of WML(-)SI(-) after adjusting for age; 95%CI, 95% confidence interval.

DHT, long-term diastolic hypertension; ISHT, long-term isolated systolic hypertension; HISTORY, with a history of hypertension and appropriate levels of blood pressure; HT, hypertension.

### Multivariate analysis

In order to adjust the effects of confounding factors associated both with blood pressure and cerebrovascular change in the aging brain, multiple logistic regression was used to analyze effects of long-term blood pressure. The effect of long-term blood pressure on WML was assessed independently from SI. The presence of WML was regarded as a dependent variable, and adjusted odds ratio of each subtype of long-term blood pressure was estimated in comparison with the reference group. Age was compulsorily retained in the model since it’s considered to be the most important confounder. Among other factors nominated in the model, smoking habit showed significance (p < .20), and remained in the final model by stepwise selection (Table 6). In the final model, DHT had a significantly high risk for WML, the estimated OR was 7.1 (95%CI 2.4-21.6). However, ISHT and HISTORY were not significantly associated with the presence of WML (adjusted OR = 1.3, 0.8, for ISHT, HISTORY).

**Table 6.** Effects on WML of hypertension subtypes and other risk factors: multivariate logistic regression model.

	Multivariate model	

	Adjusted OR	95%CI
DHT	7.1	2.4 - 21.6
ISHT	1.3	0.5 - 3.3
HISTORY	0.8	0.04 - 4.5
Higher age	1.1	0.9 - 1.2
Smoking	3.1	1.3 - 7.2

DHT, long-term diastolic hypertension; ISHT, long-term isolated systolic hypertension; HISTORY, with a history of hypertension and appropriate levels of blood pressure.

Stepwise selection was used for confounding factors; sex, lifestyle, and medical history.

Hypertension subtypes and age were compulsorily remained in the model, confounding factors that showed p< .20 were retained in the model.

Effect of long-term blood pressure on SI was assessed independently from WML by the same method as for the effect on WML (Table 7). Among the factors considered in the analysis, smoking habit and heart disease remained in the final model (p < 0.20). After adjusting for those factors, the final model showed that not only DHT but also ISHT had a significantly high risk for SI. The adjusted odds ratios were 7.2 (95%CI 2.7-19.4) for DHT, 2.3 (1.1-4.9) for ISHT, and 3.3 (0.9-10.3) for HISTORY.

**Table 7.** Effects on SI of hypertension subtypes and other risk factors: multivariate logistic regression model.

	Multivariate model	

	Adjusted OR	95%CI
DHT	7.2	2.7 - 19.4
ISHT	2.3	1.1 - 4.9
HISTORY	3.3	0.9 - 10.3
Higher age	1.0	0.9 - 1.2
Smoking	2.1	0.9 - 4.4
Heart disease	1.8	0.8 - 3.6

DHT, long-term diastolic hypertension; ISHT, long-term isolated systolic hypertension; HISTORY, with a history of hypertension and appropriate levels of blood pressure.

Stepwise selection was used for confounding factors; sex, lifestyle, and medical history.

Hypertension subtypes and age were compulsorily remained in the model, confounding factors that showed p< .20 were retained in the model.

## DISCUSSION

Hypertension has been found to be the most important risk factor of cerebrovascular changes, and significant associations of the changes were shown with self-reported histories of hypertension^1^^, ^^2^^)^, cross-sectionally measured blood pressure^1^^)^, and “prevalent hypertension” that included subjects who were previously identified as hypertensives^14^^)^. However, there remains a question whether the variability of blood pressure can affect the relationship between hypertension and cerebrovascular changes. Yamamoto et al. studied 24-hour ambulatory blood pressure among 105 lacunar stroke patients and followed them up in order to assess the difference in the occurrence of WML, SI, and symptomatic stroke^12^^)^. They found that in those who showed both WML and SI on MRI, nighttime SBP and DBP were significantly higher and nocturnal fall was significantly smaller than subjects without WML or SI. Few studies have assessed blood pressure levels in longitudinal studies, especially in the community-based healthy elderly. Thus, the present study investigated whether long-term blood pressure influenced the occurrence of cerebrovascular changes detected on CT among community-dwelling healthy elderly people.

One of the major findings of the present study was that long-term diastolic hypertension (DHT) had the highest risks of cerebrovascular changes on CT in the final multivariate model after adjusting for confounding factors (adjusted OR = 7.1, 7.2, for WML, SI). The age-adjusted models also showed that DHT had very high odds ratios among subjects with WML(+)SI(-), WML(-)SI(+), and WML(+)SI(+). In agreement to our results, a community-based study showed that the OR of WML for SBP was lower than the OR for DBP in the 65-74 year age group^1^^)^, crosssectionally. Studies for SI showed that mean 24-hour SBP and DBP were significantly higher in subjects with multiple lacunar lesions in asymptomatic elderly subjects^10^^)^. DBP was also higher in the silent infarction group detected in the community-based autopsy series in Japan^16^^)^. Our longitudinal associations should be stronger than cross-sectional associations as was reported in a study of coronary heart disease^17^^)^, and the repetitive measurements of blood pressure confirm the association. Among studies that assessed individual effects of SBP and DBP, however, some investigators showed that the effects of SBP are stronger in its association with WML than that of DBP^14^^, ^^18^^)^.

The second finding is that long-term isolated systolic hypertension (ISHT) was significantly associated with SI (adjusted OR = 2.3) but not with WML. Isolated systolic hypertension is frequently found in the elderly. It is related to cardiac diastolic function and carotid stenosis^19^^, ^^20^^)^, and antihypertensive treatment on isolated systolic hypertension reduced the incidence of stroke^21^^)^. Our finding suggests the importance of prevention of isolated systolic hypertension in the elderly because of its association with silent cerebral infarction. In addition, subjects with a history of hypertension and appropriate levels of blood pressure over ten years (HISTORY) was likely to be associated with SI , though not significant (adjusted OR = 3.3, 95%CI 0.9-10.3). Their blood pressure had stayed low when they were in their 60s and 70s because of pharmacological or non-pharmacological treatment. It is most likely that prevention and control of hypertension started from a younger age can prevent silent cerebral infarction in the elderly.

Another remarkable point is that the association of ISHT to WML was not significant. Although the pathogenesis of WML is incompletely understood, similar underlying mechanisms (small-vessel vasculopathy) for WML and SI were suggested by several studies. WML was associated with vascular risk factors^1^^, ^^18^^, ^^14^^)^, the presence of SI^6^^)^, and cerebral hypoperfusion^22^^, ^^23^^, ^^24^^, ^^25^^)^. On the other hand, the etiology of WML seems to be related to a specific type of cerebral ischemia^26^^)^; some investigators found no definite relation between periventricular white matter lesions and arteriosclerosis^27^^)^. WML also correlates with decreased myelin content, loss of ependymal cell layer and reactive gliosis at the tip of the frontal horns^28^^)^, which suggests, in addition to the ischemic mechanism, relationship with cerebrospinal fluid circulation or cerebral edema^26^^)^. Our results showing significant associations of hypertension, higher age, and smoking with WML and SI support the proposed mechanism of cerebral vasculopathy, and the difference observed in the effects of ISHT between WML and SI suggests that those changes have different underlying mechanisms.

Unexpectedly, the measures for variation of long-term blood pressure such as coefficient of variation did not seem to have associations with cerebrovascular changes. In contrast, short-term blood pressure variability, studied using a non-invasive 24-hour blood pressure recorder, seemed to influence the occurrence of cerebrovascular changes in the elderly. Both absence of nocturnal fall in SBP^11^^, ^^12^^)^ and extreme nocturnal fall^13^^)^ seemed to contribute to more widespread ischemic changes in the cerebral white matter. Time analysis would be better to assess blood pressure variability over ten years; however, it could not be performed because our community-based sample had variations in the frequency of subjects’ blood pressure measurement times (range; 3 to 10 times). Therefore variability of long-term blood pressure was measured only by the within-subject coefficient of variation (CV) and gain speed.

A limitation of this study was the lack of information about the presence of cerebrovascular changes at the baseline examination, and odds ratios found in our study were prevalence odds ratios. If our study included baseline information on cerebrovascular changes, the conclusion about the causal-effect relationship would be more definite. However, we confirmed the relationship between long-lasting hypertension and cerebrovascular changes. Considering that elderly demented patients with cerebrovascular changes had lower blood pressure in a Swedish longitudinal study^29^^)^, the reverse possibility, that cerebrovascular changes caused high blood pressure, seems unlikely, and it is reasonable to regard long-term hypertension as a risk factor of cerebrovascular changes. The other limitation is that the effect of treatment of hypertension could not be analyzed because the information on treatment obtained by interviewing the elderly subjects was unclear and with many missing values. The third point is that since our study is focused in subclinical changes among so-called “healthy” people or independently living elderly persons, many of the disabled persons did not respond to cranial CT examination, and as a result, this reduced the response rate to 44%. Our data among the elderly population showed, however, the relationship between the subclinical aspects of the aging brain and long-term blood pressure.

The present study suggests several clinically important points to improve the quality of life in the elderly population. Population-based studies for the elderly showed that WML were associated with cognitive decline^3^^, ^^4^^)^ and neurological damage^6^^)^. Elderly subjects with SI were also found to have significant psycho-neurological deficits^5^^, ^^7^^)^, and had high risks for clinical stroke^8^^)^. Furthermore, elderly patients with major depression had significantly more SI(s)^30^^)^. Future research will be needed to ascertain whether these changes would predict prognosis, and whether prevention of hypertension would protect the aging brain from functional deterioration.

In conclusion, elderly subjects with long-term diastolic hypertension had the highest risks of cerebrovascular changes, and long-term isolated systolic hypertension had the significantly high risk of silent infarction, though not of white matter lesions. These results suggest the possibility of different underlying mechanisms for WML and SI. However, both types of hypertension should be prevented in order to improve quality of life of the elderly.
